# Biomineralization and Bioaccumulation of Europium by a Thermophilic Metal Resistant Bacterium

**DOI:** 10.3389/fmicb.2019.00081

**Published:** 2019-01-30

**Authors:** Maleke Maleke, Angel Valverde, Jan-G Vermeulen, Errol Cason, Alba Gomez-Arias, Karabelo Moloantoa, Liza Coetsee-Hugo, Hendrik Swart, Esta van Heerden, Julio Castillo

**Affiliations:** ^1^Department of Microbial, Biochemical and Food Biotechnology, University of the Free State, Bloemfontein, South Africa; ^2^Institution of Groundwater Studies, University of the Free State, Bloemfontein, South Africa; ^3^Department of Physics, University of the Free State, Bloemfontein, South Africa; ^4^iWATER Solutions, Bloemfontein, South Africa

**Keywords:** biomineralization, intracellular Eu bioaccumulation, rare earth metals, thermophile, *Thermus scotoductus* SA-01

## Abstract

Rare earth metals are widely used in the production of many modern technologies. However, there is concern that supply cannot meet the growing demand in the near future. The extraction from low-grade sources such as geothermal fluids could contribute to address the increasing demand for these compounds. Here we investigated the interaction and eventual bioaccumulation of europium (Eu) by a thermophilic bacterium, *Thermus scotoductus* SA-01. We demonstrated that this bacterial strain can survive in high levels (up to 1 mM) of Eu, which is hundred times higher than typical concentrations found in the environment. Furthermore, Eu seems to stimulate the growth of *T. scotoductus* SA-01 at low (0.01–0.1 mM) concentrations. We also found, using TEM-EDX analysis, that the bacterium can accumulate Eu both intracellularly and extracellularly. FT-IR results confirmed that carbonyl and carboxyl groups were involved in the biosorption of Eu. Infrared and HR-XPS analysis demonstrated that Eu can be biomineralized by *T. scotoductus* SA-01 as Eu_2_(CO_3_)_3_. This suggests that *T. scotoductus* SA-01 can potentially be used for the biorecovery of rare earth metals from geothermal fluids.

## Introduction

Rare earth metals are essential for the production of modern devices like solar cells, mobile phones and computers, as well as for biomedical applications (Yu et al., 2009; Bouzigues et al., 2011; Das and Das, 2013; Zhuang et al., 2015). For example, europium (Eu) luminescent complexes are excellent probes for several biological and biomedical applications such as organic light-emitting diode, sensing and targeting specific DNA structures, bioimaging, melamine detection in milk products, and cellular imaging (Dutta et al., 2016; Silva et al., 2017). Thus, there is an ever increasing demand for rare earth metals due to the sheer scale and the rapid evolution of the biotechnological market. Accordingly, novel sources for viable rare earth metal supply have been explored, among them metal-rich hydrothermal fluids (Wood and Shannon, 2003; Lo et al., 2014; Charrier and Ajo-Franklin, 2017). Indeed, elevated concentrations (20–1133 nmol/kg) of rare earth metals have been detected in geothermal waters of the Yellowstone National Park (Lewis et al., 1998).

Recovery technologies for these types of metals include chemical precipitation, chemical coagulation and ion exchange, among others (Mahmoud et al., 2008; Xie et al., 2014; Gunatilake, 2015; Khawassek et al., 2015). For example, chemical precipitation is widely used for metal recovery from inorganic liquid solutions (Gunatilake, 2015). Metals can be easily precipitate by the addition of precipitant agents or by pH adjustment (Mahmoud et al., 2008). However, this process requires a large amount of chemicals, which produce large amounts of sludge (Khawassek et al., 2015). In contrast, biological approaches (e.g., bioaccumulation and biomineralization) are more cost effective and environmentally friendly (Volesky, 2001, 2007; Diniz and Volesky, 2005). Furthermore, they seem to perform very well to recover metal ions from very dilute solutions with moderate to low grade of rare earth metals, which is a common feature of geothermal fluids (Das et al., 2010; Lo et al., 2014). Nevertheless, most of these studies have been performed with mesophilic microorganisms.

Thermophilic and hyper thermophilic bacteria and archaea, such as chemoautotrophic sulfur reducer and oxidizers, also interact with metals (Hetzer et al., 2006; Babák et al., 2012; Jiang et al., 2012; Naik and Furtado, 2017). For instance, a comparative investigation on the uptake of heavy metals (Cd^2+^, Cu^2+^, Co^2+^, and Mn^2+^) in *Geobacillus thermantarcticus* and *Anoxybacillus amylolyticus* showed high affinity of metals for the cell envelope (Özdemir et al., 2013). On a dry weight basis, *G. thermantarcticus* was able to bind higher amounts of Cd and Mn more than *A. amylolyticus*. In general, the microbial binding capacity of metals is approximately on the order of 10^-5^ to 10^-3^ mol metal g^-1^ (dry weight) microbe, which compares to the binding capacities of commercial ion exchangers (Vijayaraghavan and Yun, 2008). Yet, to our knowledge, only one study using *Geobacillus stearothermophilus* as a biosorbent has recently investigated how thermophilic bacteria interact with rare earth metals (Charrier and Ajo-Franklin, 2017). Here we report the bioaccumulation and biomineralization of Eu by *Thermus scotoductus* SA-01, which was isolated from fissure water sampled at a depth of 3.2 km (Mponeng Gold Mine, South Africa) (Kieft et al., 1999). The organism is of interest because of its ability to interact with a variety of metals (Kieft et al., 1999; Opperman and van Heerden, 2007; Cason et al., 2012; Erasmus et al., 2014) under thermophilic conditions.

## Materials and Methods

### Cultivation Conditions

Unless stated otherwise, *T. scotoductus* SA-01 was cultivated under anaerobic conditions in complex organic media TYG (5 g/L Tryptone; 3 g/L Yeast Extract, and 1 g/L Glucose, pH 7.0) at 65°C on a rotary shaker (160 rpm). Cell concentrations were determined by extrapolating OD_600nm_ to dry biomass values using a calibration curve.

### Tolerance to Europium

*Thermus scotoductus* SA-01 cells were grown to mid-exponential growth phase (OD_600nm_ = 0.8), inoculated (1:10 dilution, approximately 0.06 g/L dry weight) into fresh TYG medium containing Eu (0, 0.01, 0.05, 0.1, 0.5, 1, and 2 mM) and grown for 24 h. Differences in bacterial growth between cultures were monitored spectrophotometrically (OD_600nm_) taking samples at 2 h intervals. The experiment was performed in triplicate.

### Removal of Europium

A standardized cell suspension (0.06 g/L dry weight) was used as an inoculum to initiate growth with 0.5 mM of Eu^3+^. After recording the optical density, 1 mL samples were centrifuged (6,000 ×*g*; 5 min) and the removal of Eu was evaluated by monitoring the decrease in total Eu^3+^ concentration in the media over time using the arsenazo-III method (Uhrovčík et al., 2013). Briefly, samples (0.5 mL) were added to 1 mL of a 0.1 M potassium hydrogen phthalate buffer solution, followed by 0.4 mL of the 0.05% chromogenic reagent dissolved in water. The reaction mixture was filled with deionized water to a final volume of 5 mL and mixed thoroughly. Eu^3+^ was quantified using a calibration curve relating Eu^3+^ concentration to absorbance at 655 nm (0.998 correlation coefficient) measured using a GENESYS 5 (Thermo Fisher Scientific, United States) spectrophotometer. Negative controls were used to assess abiotic Eu^3+^ removal.

### Cellular Distribution of Europium

The accumulation of Eu^3+^ by different subcellular fractions of *T. scotoductus* SA-01 was evaluated using the methodology described by Gaspard et al. (1998). Briefly, cells exposed to Eu^3+^ were harvested by centrifugation (6,000 ×*g*; 15 min; 4°C) and approximately 1 g of cells washed with 20 mM MOPS-NaOH buffer (pH 7.0). Spheroplasts were prepared by resuspending ∼1 g wet weight xperiment when concecells in 20 mL of buffer containing 25% (w/v) sucrose. Lysozyme was added to a final concentration of 0.1% (w/v) and slowly mixed on a tube roller mixer for 20 min in order to degrade the cellular wall. EDTA (pH 8.0) was added to a final concentration of 5 mM to the lysis buffer and slowly shaken for additional 20 min. Magnesium chloride (MgCl_2_) was added to a final concentration of 13 mM and the suspension was further shaken for 20 min. Separation of spheroplast from the periplasmic fraction was achieved by centrifugation (20,000 ×*g*; 30 min; 4°C). Spheroplasts were resuspended in 10 mL of 20 mM MOPS-NaOH buffer (pH 7.0).

To obtain the membrane and cytoplasmic fractions, cells were disrupted by ultrasonic treatment (6 repeats, 100 W, 30 s on ice) with a Branson Sonic Power Sonifier Cell Disruptor B-30 (Danbury, United States). Cell debris was removed by centrifugation (4,000 ×*g*; 10 min; 4°C). The crude extract (supernatant) was subsequently centrifuged (100,000 ×*g*; 90 min; 4°C), yielding a cytoplasmic fraction containing soluble proteins (supernatant) and a membrane fraction (pellet). The latter fraction was resuspended in MOPS–NaOH buffer (20 mM, pH 7.0) and the concentrations of Eu in all fractions were immediately determined using the arsenazo-III method.

### Scanning and Transmission Electron Microscopy

Electron microscopy was utilized to investigate the sorption and/or accumulation of Eu. *T. scotoductus* SA-01 cells exposed to 0.5 mM Eu were harvested by centrifugation (6,000 ×*g*; 15 min; 4°C). For SEM, the cells were fixed in 2.5% (v/v) glutaraldehyde, and dehydrated. Thereafter, the cells were critical point dried, mounted on metal stubs, coated with gold and analyzed using a JSM-7800F thermal field emission scanning microscope (FE-SEM) coupled with Oxford Aztec 350 X-Max80 energy-dispersive X-ray (EDX) analysis (Oxford Instruments, United Kingdom). For TEM, the cell pellets were subjected to fixation, dehydration, and polymerization. Thin sections (0.2 μm) were cut and trimmed with an ultra-microtome UM7 (Leica Microsystems, Germany) and collected on copper grids. Transmission electron micrographs were taken with a Philips CM100 (FEI, United States) coupled with an Oxford X-ray analyzer coupled with energy dispersive X-ray (EDX) spectrum (JSM-7800F) (Oxford Instruments, United Kingdom).

### Fourier Transform Infrared (FT-IR) Spectroscopy

Fourier transform infrared spectroscopy was used to elucidate functional groups interacting with Eu. After centrifugation as above, the bacterial cells were dried overnight by lyophilization under vacuum and analyzed using a Bruker Tensor 27 model (Bruker, Germany). The spectral analysis was done in the mid IR region (500–4000 cm^-1^) with 16 scan speed. Peaks were identified based on previously reported data.

### High-Resolution X-Ray Photoelectron Spectroscopy (HR-XPS)

High-resolution X-ray photoelectron spectroscopy was used to determine both Eu oxidation state and neoformed mineral phases. HR-XPS was obtained with a PHI 5000 Versaprobe system (Physical Electronics, United States). Briefly, after incubation following standard conditions, bacterial cells were harvested by centrifugation (6000 ×*g*; 15 min; 4°C), the pellets were dried under vacuum by lyophilization, embedded on a carbon tape and then analyzed in a vacuum chamber. A low energy Ar ion-gun and low energy neutralizer electron-gun were used to minimize charging on the surface. A 100 μm diameter monochromatic Al Kα x-ray beam (hν1/4 1486.6 eV) generated by a 25 W, 15 kV electron beam was used to analyze the different binding energy peaks. The pass energy was set to 11 eV giving an analyzer resolution of 0.5 eV. Multipack version 8.2 software (Ulvac-PHI, Inc., Japan) was utilized to analyze the spectra to identify the chemical compounds present and their electronic states using Gaussian–Lorentz fits.

## Results

### Tolerance to Europium

**FIGURE 1:**
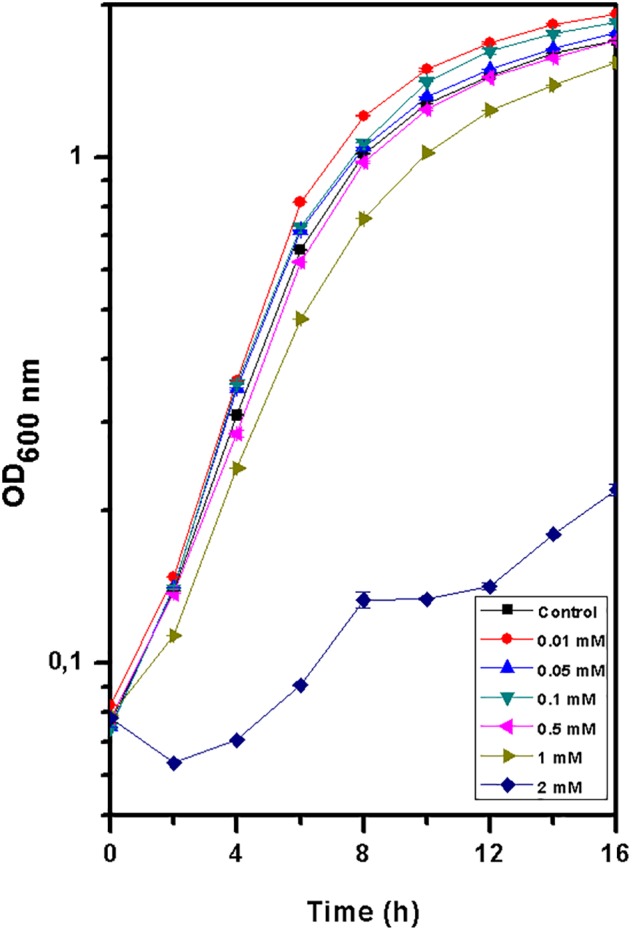
Growth curve of *Thermus scotoductus* SA-01 in TYG media over a 16 h period. Symbols indicate the mean value of OD_600nm_ samples. Standard deviations are included but are negligible.

The growth of *T. scotoductus* SA-01 in TYG medium was identical when exposed to concentrations between 0.01 and 1 mM of Eu^3+^ (Figure 1 and Table 1), although slower growth was observed at the beginning of the experiment when concentration of Eu were >0.5 mM. In contrast, no growth was observed at 2 mM of Eu, suggesting that at this concentration Eu is toxic for this bacterium.

**Table 1:** Specific growth rate and doubling time values for *Thermus scotoductus* SA-01 grown in different Eu (0, 0.01, 0.05, 0.1, 0.5, 1, and 2 mM) concentrations.

Concentration	μMax (h^-1^)	td (h)
Control (0 mM)	0.34 ± 1.9*E*-04	2.14 ± 5.4*E*-03
0.01 mM	0.39 ± 3.5*E*-04	1.81 ± 3.2*E*-03
0.05 mM	0.38 ± 2.4*E*-04	1.82 ± 1.5*E*-03
0.1 mM	0.37 ± 1.1*E*-04	1.84 ± 1.2*E*-03
0.5 mM	0.35 ± 2.3*E*-04	2.04 ± 6.4*E*-03
1 mM	0.31 ± 9.2*E*-04	2.47 ± 7.2*E*-03
2 mM	BD^∗^	–


^∗^BD, below detection limit.

### Removal of Europium

**FIGURE 2:**
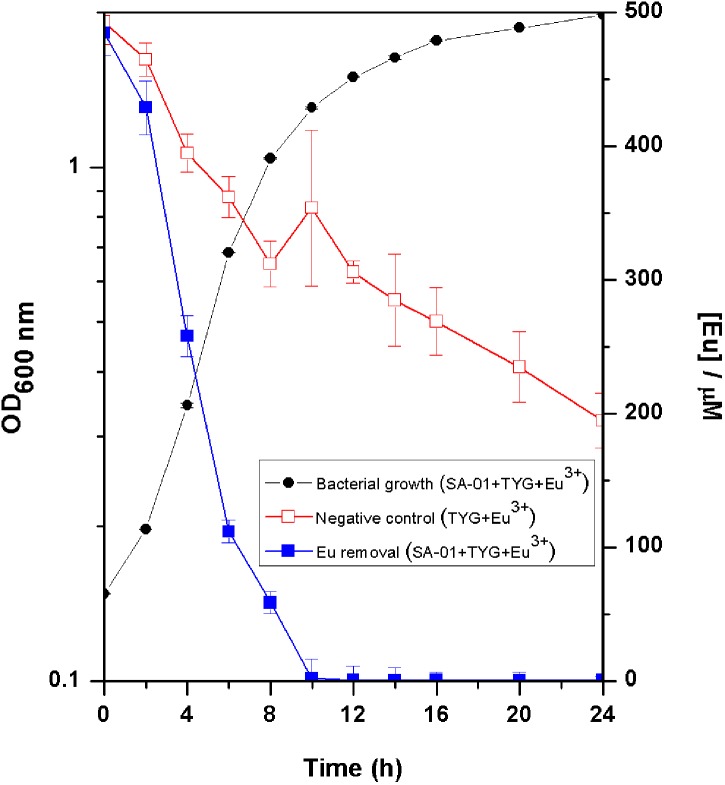
Growth curve of *T. scotoductus* SA-01 supplemented with 0.5 mM Eu^3+^ in TYG media. Error bars indicate standard deviation, while symbols indicate mean values.

Europium was totally removed by *T. scotoductus* SA-01 within 10 h of incubation during the exponential growth phase (Figure 2). We note that Eu precipitation also took place in the negative controls, but in lower amount than in the presence of *T. scotoductus* SA-01, likely due to the change in physicochemical parameters other than pH, as pH did not vary significantly and kept neutral until the end of the experiments (from 7 to 6.5 on average).

### Bioaccumulation of Europium

**FIGURE 3:**
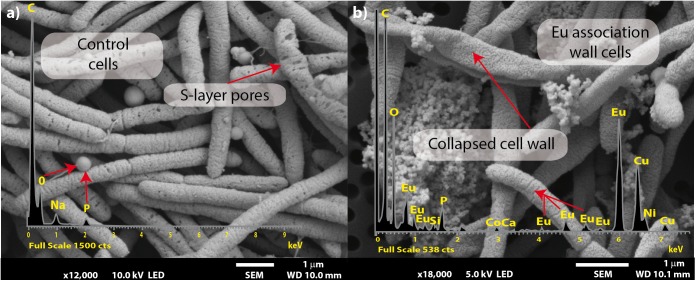
Scanning electron microscopy micrographs of *T. scotoductus* SA-01. Bars indicate the scale as micrometers and red arrows indicate EDX spectra. Control **(a)** and Eu amended cells **(b)**.

Scanning electron micrographs showed that most of the cells exposed to Eu were similar in morphology to those unexposed (Figure 3). Several collapsed cells were found in the preparations but with a similar rod-shaped form as those of the control cells (Figure 3b). Metal precipitates were also observed and electron dispersion X-ray (EDX) spectroscopy analyses revealed that the precipitates were mainly composed of Eu, P, C, and O (inset Figure 3b).

**FIGURE 4:**
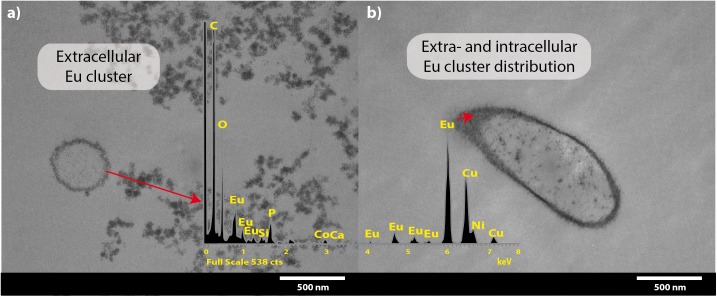
Transmission electron microscopy micrographs of *T. scotoductus* SA-01 amended with 0.5 mM Eu. White bars indicate the scale in nanometers and red arrows show the location and distribution of Eu (shown as electron-dense granules). Europium biosorption **(a)** and intracellular bioaccumulation **(b)**. Insets show energy-dispersive X-ray spectra.

Transmission electron microscopy coupled to EDX spectra analysis corroborated that most Eu deposits accumulated on the cell surface (Figure 4a,b), although Eu precipitates were also intracellularly accumulated (Figure 4b). The presence of the intense copper (Cu) peak is attributed to the copper grid used for sample collection.

Similar results were obtained after the separation of the different subcellular fractions. Approximately 78% of the Eu^3+^ retained (0.36 mM out of 0.5 mM) by the bacterium was found on the cell surface, 17% on the cytoplasmic membrane and 5% in the cytoplasm. We did not detect any Eu^3+^ in the periplasmic fraction.

### Surface Characterization

**FIGURE 5:**
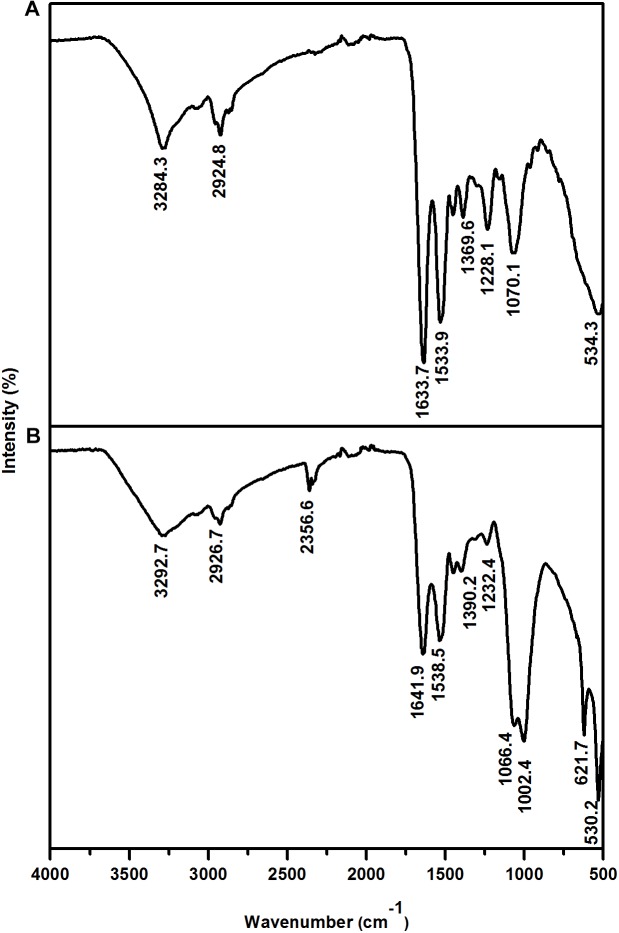
Fourier transform infrared spectrum of *T. scotoductus* SA-01. Control **(A)** and cell cultures supplemented with Eu **(B)**.

The interaction between the cell wall and Eu was further assessed by Fourier transform infrared (FTIR) analysis. The FTIR spectra were in the range of 500–4000 cm^-1^ (Figure 5 and Table 2). Prominent peaks in the loaded biomass were observed at 621.7, 1002.4, 1066.4, and 2356.6 cm^-1^. While, the intensity of some peaks (at 1228.1, 1538.5, 1641.9, 2926.7, and 3292.7 cm^-1^) in the loaded biomass was substantially lower than the unloaded biomass. The peak stretching and intensity demonstrate a change in the amount of the functional group associated with the molecular bond. Whereas a shift in peak position demonstrates the hybridization state in the molecular bond has changed. The spectra showed a distinctive peak at 621.7 cm^-1^ attributed to PO_4_^3-^ in the loaded biomass, which is absent in the control samples. Peaks attributed to organic phosphate and C-PO_3_^2-^ (1002.4 and 1066.4 cm^-1^), CO_2_ (2356.6 cm^-1^) and alkyl chain bands (around 2850–2955 cm^-1^) were also observed. Low intensity peaks were also noted, for instance, peaks between 1056 and 1233 cm^-1^, which are attributed to P-O of C-PO_3_^2-^ moiety region and P = O, as well as, lower intensity peaks around 1400 and 1600 cm^-1^, which contribute to the amide I and II regions of proteins, were reduced in cells binding Eu. The amide II region consists of N–H bending and C–N stretching vibrations close to the region of 1520–1550 cm^-1^. While, amide I is usually the region at 1633 cm^-1^ but in the experiments shifted to 1641 cm^-1^, which was caused by C = O stretching. Overall, the spectra indicated that the interaction occurs mainly through the phosphate, carboxyl and carbonyl of amide groups.

**Table 2:** Assignment of FTIR derived peaks to functional groups of *T. scotoductus* SA-01.

Wavenumber (cm^-1^)	
Control	Europium	Assignment	Reference
1010.1	1002.4	Organic phosphate	Hosomomi et al., 2013
–	1066.4	P-O of C-PO_3_^2^**^-^**	Hosomomi et al., 2013; Oves et al., 2013
1228.1	1232.4	P = O phosphodiester	Kamnev et al., 2002; Hosomomi et al., 2013; Oves et al., 2013
1369.6	1390.2	COO**^-^** (carboxyl) and C-O-C	Emmanuel et al., 2011; Hosomomi et al., 2013
1533.9	1536.5	Amide II; N-H and C-N group	Adochitei and Drochioiu, 2011; Hosomomi et al., 2013; Oves et al., 2013
1633.7	1641.9	Amide I; C = O group (carbonyl)	Adochitei and Drochioiu, 2011; Hosomomi et al., 2013; Oves et al., 2013
2924.8	2926.7	C-H stretching and alkyl group	Hosomomi et al., 2013; Oves et al., 2013
3284.3	3292.7	O-H of carboxyl stretching/N-H stretching	Kamnev et al., 2002; Emmanuel et al., 2011


### Biomineralization of Europium

**FIGURE 6:**
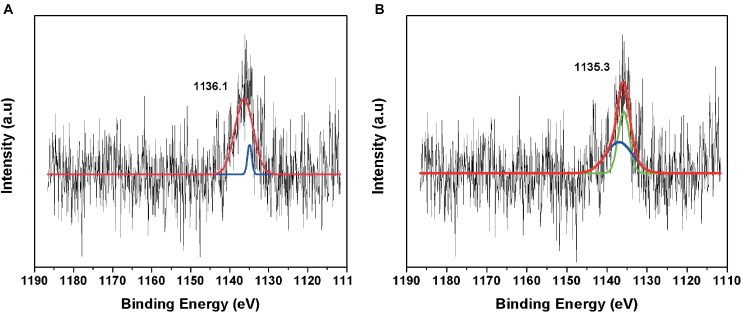
High-resolution XPS spectrum of **(A)** negative control amended with Eu and **(B)** minerals formed in TYG media amended with Eu and *T. scotoductus* SA-01.

The fitted curve of the HR-XPS spectra indicated two major peaks at 1135.1 and 1131.6 eV (Figure 6). The HR-XPS analysis revealed that the Eu was in the 3+ oxidation state. According to Mercier et al. (2006), the 1135.1 eV peak was identified as Eu carbonate [Eu_2_(CO_3_)_3_].

## Discussion

Rare earth metals, including Eu, have recently been found to play an important role in the biology of different bacteria (Pol et al., 2014; Jahn et al., 2018). Here we found that Eu promotes the growth of *T. scotoductus* SA-01 at low concentration (up to 0.1 mM), while it is detrimental at high concentrations (>0.5 mM). This is in accordance with the results reported by Azabou et al. (2007) in *Desulfomicrobium* species, a mesophilic bacterium. Similarly, Pol et al. (2014) showed that the growth of *Methylacidiphilum fumariolicum* SoIV (thermophilic) was affected by rare earth metals. Comparatively, the tolerance of *T. scotoductus* SA-01 is higher than that of other bacterial strains reported to tolerate rare earth metals. For example, *Bacillus* sp. W-28 and *S. acidiscabies* W-12 (both mesophilic) can tolerate 0.05 and 0.2 mM of La, respectively (Haferburg et al., 2007). Conversely, *T. scotoductus* SA-01 tolerance is lower than that reported for *K. vulgare* (mesophilic) which is able to tolerate 5 mM of La (Lyu et al., 2014). In general, the toxicity of metals in bacteria results from the displacement or substitution of essential elements from cellular sites and due to the blocking of functional groups of important biochemical molecules (Cason et al., 2012). For instance, rare earth metals can replace calcium ions in the binding sites of nucleases, affecting bacterial growth (Balasubramanian et al., 2014).

Rare earth metals substantially change bacterial cell walls particularly those of gram-negative bacteria (Peng et al., 2004; Chen et al., 2010, 2012). Likewise, we also found morphological changes in the bacterial cells treated with Eu. In general, the cell wall has high affinity for metals in solution (Mishra et al., 2010; MacHalová et al., 2015), which bind to cell surface receptors such as S-layer proteins and other organic molecules (Borrok et al., 2007; Hosomomi et al., 2013; MacHalová et al., 2015). The SEM and TEM micrographs and cellular fractionation showed that Eu accumulates (in decreasing order), on the cell surface of the bacterial envelopes, in the membrane and in the cytoplasm, but not in the periplasmic space. These results are in contrast to what was observed in *E. coli*, which was able to accumulate rare earth metals in the periplasmic space (Bayer and Bayer, 1991). We did not investigate the molecular mechanism that allow Eu to enter the cytoplasm, but previous studies using this bacterium and other metals (i.e., U and Au) seem to indicate that ABC transporters (Cason et al., 2012; Erasmus et al., 2014) may play a role. On the other hand, the intracellular accumulation of Eu might be mediated by PolyP metabolism. Indeed, transmission electron microscopy and EDX analysis showed electron dense granules in the cytoplasm composed of Eu and phosphate. Furthermore, *T. scotoductus* SA-01 harbors polyphosphate kinases (ppk) and exopolyphosphatases (ppx) genes, which are responsible for the synthesis and degradation of PolyP, respectively. PolyP is often involved in metal accumulation and detoxification in bacteria (Rao and Kornberg, 1996; Kuroda et al., 1999; Nikel et al., 2013) as a defense mechanism against environmental stress. Overall, the data seem to indicate that there is a rapid phase of metal binding to the cell surface (biosorption) that is followed by a slower phase of metal bioaccumulation into the cell.

We further investigated which functional groups could be involved in the biosorption of Eu. The FT-IR results showed that peaks associated with functional groups such as phosphates (PO_4_), carboxyl (COOH) and carbonyl (C = O) of amide groups, commonly found as organic molecules released by microorganism, become more evident after the incubation with Eu (Benzerara et al., 2005). These functional groups as well as others (e.g., aldehyde, hydroxyl, ketone) are commonly involved in the biosorption of metals in mesophilic bacteria (Madrid and Camara, 1997; Saleem et al., 2008), but few reports are available on thermophiles (Özdemir et al., 2013). Elements such as Ca^2+^ can react with COOH and C=O groups to form various chelate complex [Ca_x_^2+^(CO)_y_]^n^ (Qian et al., 2010). Interestingly, Europium (Eu^3+/2+^) has similar ionic charge and radius to Ca^2+^, which facilitate the replacement of Ca by Eu in mineral structures (Homer and Mortimer, 1978; Hellebrandt et al., 2016). This suggests the biomineralization of Eu as neoformed mineral complex [Eu_x_^+^(CO)_y_]^n^ on the cell wall. Indeed, HR-XPS analysis demonstrate that the phase minerals bound to the cell surface of *T. scotoductus* SA-01 were Eu_2_(CO_3_)_3_. However, a significant drawback to this technique is that it only provides information with regards to surface binding of Eu (Kumar et al., 2015). Usually, reduction of metals leads to intracellular bioaccumulation as it was observed in bacterium *Paracoccus denitrificans* interaction with Cu (Su et al., 2015). Therefore, it is possible that a fraction of Eu accumulated intracellularly might exist in the divalent state.

**FIGURE 7:**
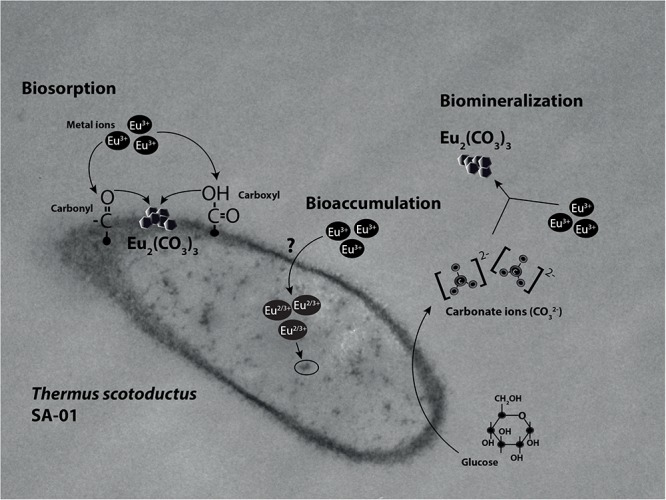
Illustration of the interaction mechanisms of *T*. *scotoductus* SA-01 with Europium.

Microorganisms can also induce the precipitation of minerals by modifying the conditions of their surrounding microenvironments (De Muynck et al., 2013; Sánchez-Román et al., 2015; Zhu and Dittrich, 2016). Under neutral to alkaline pH, the carbon dioxide produced by respiration reacts easily with OH^-^ radicals leading to the formation of carbonate minerals. For example, Sánchez-Román et al. (2015) reported that the increase in CO_3_^2-^ induced Fe-carbonate mineralization in *Tessarococcus lapidicaptus*. Several studies have also reported on the external precipitation of Ca-carbonate by *Cyanobacteria* (Obst et al., 2006; Kamennaya et al., 2012; Benzerara et al., 2014). Altogether, it seems that the presence of *T. scotoductus* SA-01 can induce the biomineralization of Eu in two different ways: (1) by modifying the conditions of its surrounding microenvironments and/or (2) acting as nucleation sites (Figure 7).

## Conclusion

Our findings provide information on how *T. scotoductus* SA-01 interacts with Eu under thermophilic conditions. This is important because temperature is a limiting factor when exploring metal recovery from geothermal fluids by biological processes and for the use of bacteria in other industrial applications. We conclude that *T. scotoductus* SA-01 can be employed for the biorecovery of Eu and other rare earth metals in rare earth metal-containing carbonates.

## Author Contributions

MM, JC, JV, EC, and EvH designed the research. MM and KM performed the experiments. AG-A helped with ICP-MS analysis and data interpretation. LC-H and HS helped with HR-XPS analysis and data interpretation. MM wrote the first draft of the manuscript. MM, AV, JC, JV, EC, KM, and EvH wrote the final manuscript. All authors read and approved the final manuscript.

## Conflict of Interest Statement

The authors declare that the research was conducted in the absence of any commercial or financial relationships that could be construed as a potential conflict of interest.
